# Distinct subtypes of kidney transplant-associated urothelial carcinomas harboring BK polyomavirus integration and aristolochic acid mutational signatures

**DOI:** 10.1016/j.bj.2026.100990

**Published:** 2026-05-23

**Authors:** Jiun-Wen Wang, Yi-Jung Li, Tzu-Hao Huang, Hsin-Hsu Wu, Hsu-Han Wang, Yang-Jen Chiang, See-Tong Pang, Abner Herbert Lim, Cedric Chuan Young Ng, Wei Liu, Jing Yi Lee, Jing Han Hong, Bin Tean Teh, Jason Yongsheng Chan, Ya-Chung Tian

**Affiliations:** a Kidney Research Center and Department of Nephrology, Linkou Chang Gung Memorial Hospital, Taoyuan, Taiwan; b Department of Medicine, Chang Gung University, Taoyuan, Taiwan; c Department of Urology, Taipei Veterans General Hospital, Taipei, Taiwan; d Department of Urology, Taiwan School of Medicine and Institute of Clinical Medicine, College of Medicine, National Yang Ming Chiao Tung University, Taipei, Taiwan; e Graduate Institute of Clinical Medical Sciences, Chang Gung University, Taiwan; f Department of Urology, Linkou Chang Gung Memorial Hospital, Taoyuan, Taiwan; g Cancer Discovery Hub, National Cancer Centre Singapore, Singapore; h Cancer and Stem Cell Biology Programme, Duke-NUS Medical School, Singapore; i Oncology Academic Clinical Program, Duke-NUS Medical School, Singapore; j Laboratory of Cancer Epigenome, National Cancer Centre Singapore, Singapore

**Keywords:** Urothelial carcinoma, Kidney transplant recipients, BK polyomavirus, Aristolochic acid, Whole-genome sequencing, Mutational signatures

## Abstract

**Background:**

Urothelial carcinoma (UC) is common in kidney transplant recipients, especially in Taiwan. Both BK polyomavirus (BKPyV) genome integration and aristolochic acid (AA) mutagenesis have been implicated in UC carcinogenesis, but their relative contributions remain unclear.

**Material and methods:**

To characterize the molecular features of UC in kidney transplant recipients, we performed whole-genome sequencing (WGS) on 19 tumor specimens and analyzed BKPyV integration sites and AA mutational signatures.

**Results:**

BKPyV genome integration was detected in 6 of 19 (32%) UC specimens, yielding a total of 21 integration sites. Chromosomes 8 and 12 exhibited higher frequencies of integration events. Among these integration sites, 76% were located within introns, 19% in intergenic regions, and one within an exon. AA mutational signatures were identified in 84% of UC specimens. An APOBEC signature was found in 89% of specimens, with high activity in two specimens from patients with polyomavirus nephropathy. Frequent somatic mutations were observed in genes related to chromatin modification [KMT2C (68%), KMT2D (53%), CREBBP (42%), ARID1A (42%)], cell-cycle regulation [TP53 (58%), NF1 (47%), ATM (32%)], and oncogenic signaling [ERBB2 (21%), PIK3CA (16%), FGFR3 (11%), HRAS (11%)]. Notably, UC specimens with BKPyV integration harbored significantly fewer mutations compared to those with AA mutational signatures.

**Conclusion:**

Our findings reveal two distinct molecular subtypes of UC in kidney transplant recipients: one characterized by BKPyV genome integration and the other by AA-associated mutagenesis. These observations suggest divergent mechanisms of UC tumorigenesis and may have implications for diagnosis and treatment in this high-risk population.

## 1 Introduction

The chance of developing post-transplant malignancies is estimated at 5-10% ten years post-kidney transplantation and over 20% beyond twenty years [[1], [2], [3]]. Several factors shared by the at-risk population may contribute to the development of such cancers, including carcinogenic substances and viral infection. In particular, kidney transplant recipients have an increased risk of developing cancers due to impaired immune surveillance and clearance of neoplastic cells.

Viral infections are responsible for 10-20% of worldwide cancers and are more prevalent in organ transplant recipients [[3], [4], [5]]. Recent studies have shown that Merkel cell polyomavirus (MCPyV), a member of the polyomaviridae family, is an oncogenic virus strongly associated with the development of Merkel cell carcinoma (MCC). The MCPyV genome is known to integrate into the human genome, leading to clonal expansion of tumor cells [6]. More recently, the association of human herpesvirus-7 (HHV-7) with angiosarcoma has been similarly described [7]. Interestingly, independent from their viral associations, both of these cancer types are also etiologically-associated with ultraviolet mutagenesis [7,8]. In this study, we investigated the relationship of viral and herbal carcinogens with upper urinary tract urothelial carcinoma (UTUC), which has a significantly higher incidence in Taiwan and predisposition in kidney transplant recipients. BK polyomavirus (BKPyV) belongs to the polymaviridae family, which can infect humans during childhood and remain latent throughout their lifetime, especially in the kidneys and urinary system [9]. It reactivates rapidly in immunosuppressed hosts such as kidney transplant recipients, leading to polyomavirus-associated nephropathy (PyVAN) [[10], [11], [12]]. The oncogenic potential of BKPyV has been demonstrated *in vitro* and *in vivo* [[13], [14], [15], [16]]. In humans, a high incidence and early onset of urinary tract cancers were observed in kidney transplant recipients with PyVAN, suggesting a correlation between BKPyV and oncogenesis [17]. Kenan et al. demonstrated the integration of BKPyV genome into the human genome in a specimen of urothelial carcinoma (UC) and a specimen of renal cell carcinoma from two kidney transplant recipients [18,19]. Recently, two studies also discovered the integration of BKPyV genome in more UC specimens of kidney transplant recipients, providing direct evidence of the oncogenic potential of BKPyV [20,21].

Other factors such as exposure to the natural compound aristolochic acid (AA), a group I human carcinogen found in Aristolochia plants, can also contribute to cancer development [22]. AA exposure is associated with rapid kidney fibrosis and AA nephropathy [23,24], as well as a high risk of urinary tract cancers, including urothelial carcinoma, due to its genotoxic mutagenic properties that form DNA adducts [25,26]. Our previous studies have shown specific AA mutational signatures in urinary tract and liver cancers, implicating its role in cancer development in the general population [[27], [28], [29], [30]]. While the use of AA-containing herbal medicine has been officially banned in Taiwan since the early 2000s, it is believed that they may still be found and consumed in the population, probably due to the lack of knowledge about the content of AA in some of the herbal plant families and the challenge to enforce the ban through online sales. AA exposure can lead to accumulation of DNA adducts and kidney transplant recipients with impaired immune function may be vulnerable to both AA and BKPyV related cancer development particularly in the urinary tract.

## 2 Materials and methods

### 2.1 Sample collection

Five fresh urothelial carcinoma (UC) specimens were obtained from Linkou Chang Gung Memorial Hospital, and fourteen frozen tissue specimens were provided by the Tissue Bank of Taipei Veterans General Hospital. Specimen collection was approved by the Institutional Review Board of Linkou Chang Gung Memorial Hospital (201801596B0C601) and the Ethics Committee of Taipei Veterans General Hospital (2020-03-007CC).

### 2.2 Whole genome sequencing

Germline whole blood and tumor DNA was extracted using QIAamp DNA mini kit (#51306, QIAGEN) and sequenced on the Illumina NovaSeq 6000 platform (NovogeneAIT Genomics, Singapore). Germline samples were sequenced at an average depth of 30x and 60x for tumor samples. The sequencing reads were aligned to the human reference genome NCBI GRC Build 37 (GRC B37) and BKPyV reference genome (NCBI: NC_001538.1) to capture viral integration sites with BWA-MEM (version 0.7.17-r1188). MarkDuplicates was used to identify potential PCR and optical duplicates and BaseRecalibrator was used for base recalibration using known sites from gnomAD (version 2.1.1). MarkDuplicates and BaseRecalibrator are both distributed as part of the Genome Analysis Toolkit (GATK, version 4.1.7.0). Details regarding mapping statistics and duplication rates are summarized in Supplementary material, [Table S1].

Variant calling was performed using Mutect2 (version 2.2) in tumor-normal paired mode. Next, FilterMutectCalls tool was used to remove potential false positives. Copy number analysis was performed in tumor-normal paired mode using ASCAT 2.5.3. TMB calculation and visualization of annotated variants were conducted as described previously [31]. Mutational signature analysis was conducted using SigProfilerExtractor (version 1.1), which extracts mutational signatures *de novo*, decomposes and assigns the mutational signatures into COSMIC (version 3.2).

To identify the presence of BKPyV from WGS, SAMtools software (version 1.11) was used to identify unmapped and soft-clipped reads not belonging to human sequences. Soft-clipped reads containing partially mapped sequences to the human genome were subsequently removed using SE-MEI extractSoftClip (version 0.1.0). Then, the filtered reads were then queried against the BKPyV genome using BLASTn (BLAST+, version 2.12.0), and matching hits with a minimum e-value of 1e-10 were kept as confident BKPyV hits.

For mapping to other viruses apart from BKPyV, unmapped reads were queried against the NCBI RefSeq viral genomic database (release 1.1, downloaded from ftp://ftp.ncbi.nlm.nih.gov/refseq/release/viral/viral.1.1.genomic.fna.gz) using BLAST (version 2.16). BLASTn queries were performed using stringent parameters: an e-value threshold of 1e-5, sequence identity 90%, and word size of 11, with results output in tabular form (outfmt 6). BLAST output tables were processed and analyzed within a Jupyter notebook environment using Biopython (version 1.80) and pandas (version 1.22). Biopython was utilized to parse the headers from the NCBI RefSeq viral genomic database, facilitating mapping between viral accession numbers and corresponding names for downstream interpretation. BLAST results were loaded into pandas DataFrames, sorted by ascending e-value and descending bitscore, and filtered to retain only the best alignment for each query sequence. Aggregation of these alignments provided a summary table reporting the number of occurrence of each identified virus across the analyzed cohort.

### 2.3 Identification of BKPyV integration sites using hybrid capture panel

A custom hybrid-capture panel was designed using the Agilent SureDesign platform (Agilent Technologies, CA, USA) to cover all exonic regions of 16 human cancer genes (*ARID1A*, *ATM*, *CREBBP*, *FAT1*, *FGFR3*, *FLT4*, *KDM6A*, *KDR*, *KRAS*, *LRRK2*, *PIK3CA*, *PLCG1*, *POT1*, *PTPRB*, *RB1*, *TP53*), as well as the complete genomes of BK virus and human herpesvirus-7 (HHV-7) (total target capture size of 483 kb). The 16 cancer genes and HHV-7 genome were included for a different project. The areas were covered with 120-bp biotinylated oligonucleotides tiled with a 20-bp overlap between adjacent probes. Germline and tumor DNA was extracted using QIAamp DNA mini kit (#51306, QIAGEN). The sequencing library was constructed using the Agilent SureSelect XT HS system (Agilent Technologies, CA, USA), a standard, high-sensitivity library preparation kit for target enrichment with single indexing. Hybrid capture was performed on individual DNA samples (100 ng), using 3-4 cycles for library amplification and quality control on the BioAnalyzer. Sequencing was performed as 2 × 150 bp reads on an Illumina NovaSeq 6000 platform (NovogeneAIT Genomics, Singapore) targeting a mean on-target depth of 2000x. To capture viral integration sites, the sequencing reads were aligned to an in-silico concatenated human reference NCBI GRC Build 37 (GRC B37) and BKPyV reference genome (NCBI: NC_001538.1) with BWA-MEM (version 0.7.17-r1188). Subsequently, GATK MarkDuplicates (GATK, version 4.17.0) were used to identify and remove potential PCR and optical duplicated reads. Possible chimeric reads spanning human and BKPyV genomes, unmapped and soft-clipped reads were filtered using SAMtools (version 1.11) for subsequent analysis. The extractSoftClip feature from SE-MEI (version 0.1.0) to filter human sequences from soft-clipped reads. These filtered reads were then mapped to the BKPyV nucleotide blast database using BLASTN (BLAST+, version 2.12), and matching hits with a minimum e-value of 1e-10 were kept for analysis of viral genome integration.

### 2.4 Polymerase chain reaction

The PCR amplification was carried out as described previously. The specific primers for BKPyV TAg (large T antigen) were 5′-ATG GAT AAA GTT CTT AAC AGG G-3′ and 5′-CTC TGA GCT ACT CCA GGT T-3’. Confirmatory nested PCR reactions were performed using primers specific for the eukaryotic translation initiation factor 2B subunit alpha (*EIF2B1)* and the solute carrier family 26 member 7 (*SLC26A7)*, as well as BKPyV sequences located on both sides of the breakpoints of viral integration into the tumor genome. The primer set for *EIF2B1* comprised 5′-CCGACCTGTATCTTCAATTCATTCA-3′, 5′-ACAACCTTAGCCTCACCCATAC-3′, and 5′-ACCCAGCTTTATTTCTATGTG-3′, while the primer set for *SLC26A7* comprised 5′-GCATAACATACTCACAGGTTC-3′, 5′-CTACTGAATTAGAGTCAGCAC-3′, 5′-TTATGAGGGAAGGAACAACAC-3′, and 5′-ATGGATAAAGTTCTTAACAGGG-3’.

## 3 Results

### 3.1 The integration of BKPyV genome into the human genome verified by whole genome sequencing (WGS)

We obtained five fresh specimens of urothelial carcinoma (UC) from four kidney transplant recipients and one non-transplant subject from Linkou Chang Gung Hospital (LCGMH), as well as 14 frozen specimens of UC from 14 kidney transplant recipients from the Tissue Bank of Taipei Veteran Hospital (TVH) [Table 1]. Matched normal DNA was obtained from adjacent non-tumor tissue for all cases except cases L2 and L3 which were from whole blood. Clinical characteristics of the five subjects from LCGMH were presented in [Table 2]. However, the clinical information of the 14 subjects from TVH was unknown because the data in those subjects were decoded. Two kidney transplant recipients at LCGMH were diagnosed with BKPyV-associated nephropathy (BKPyVAN), and, consistent with our previous study [20], these two subjects with BKPyVAN had an earlier onset of UC (47 and 74 months post-transplant) than the other two subjects without BKPyVAN (116 and 208 months post-transplant). Among the 19 UC specimens, four tested positive for TAg immunostaining, while none exhibited VP1 immunostaining [Supplementary Fig. S1]. These TAg-immunostaining positive specimens all belong to UC with BKPyV-genome integration.

**Table 1:** BKPyV genome integration and the AA mutational signature in urothelial carcinoma.

Sample	Hospital	Kidney transplant recipient	Biopsy-proven BKPyVAN	TAg/VP1 immunostaining	BKPyV genome integration	AA mutational signature
L1	LCGMH^a^	Yes	**+**	**+/−**	**+**	**-**
L2	LCGMH	Yes	**-**	**−/−**	**-**	**+**
L3	LCGMH	Yes	**+**	**+/−**	**+**	**-**
L4	LCGMH	Yes	**-**	**+/−**	**+**	**+**
L5	LCGMH	No	**-**	**+/−**	**+**	**+**
T1	TVH^b^	Yes	unknown	**−/−**	**-**	**+**
T2	TVH	Yes	unknown	**−/−**	**-**	**+**
T3	TVH	Yes	unknown	**−/−**	**-**	**+**
T4	TVH	Yes	unknown	**−/−**	**-**	**+**
T5	TVH	Yes	unknown	**−/−**	**-**	**+**
T6	TVH	Yes	unknown	**−/−**	**-**	**+**
T7	TVH	Yes	unknown	**−/−**	**+**	**-**
T8	TVH	Yes	unknown	**−/−**	**-**	**+**
T9	TVH	Yes	unknown	**−/−**	**-**	**+**
T10	TVH	Yes	unknown	**−/−**	**-**	**+**
T11	TVH	Yes	unknown	**−/−**	**+**	**+**
T12	TVH	Yes	unknown	**−/−**	**-**	**+**
T13	TVH	Yes	unknown	**−/−**	**-**	**+**
T14	TVH	Yes	Unknown	**−/−**	**-**	**+**

n = 19					6 (32%)	16 (84%)

a Linkou Chang Gung Memorial Hospital.

b Taipei Veterans General Hospital.

**Table 2:** Characteristics of kidney transplant recipients with UC.

Subjects	L1	L2	L3	L4	L5
Kidney transplant	Cadaveric	Cadaveric	Living	Living	Non-transplant
BKPyV genome integration	+	-	+	+	+
BKPyVAN	+	-	+	-	N/A
AA mutational signature	-	+	-	+	+
Herbal medicine use	-	+	-	+	+
Age	38	69	35	68	66
Sex	M	F	M	F	F
Diabetes	-	+	-	-	+
Transplant duration (months)	86	191	104	208	N/A
Serum creatinine	3.30	0.93	dialysis	1.03	0.77
eGFR	21	59	dialysis	48	75
WBC	7700	6300	7100	10400	7700
Hb	8.1	9.2	10.8	10.4	13.3
Platelet (×1000)	183	158	175	253	165
Urothelial carcinoma					
Site	UB	UB	UB	UB	UB
Staging	T1	T1	T1	T1	T1
Grade	High	High	High	High	High
Metastasis	No	Peritoneum	No	Liver/Peritoneum	Lung
Numbers of cancer recurrence (months)	1/39	7/75	2/30	9/65	3/54
Onset after transplantation (months)	47	116	74	143	N/A

N/A: not applicable.

We extracted genomic DNA from the tumor tissues and performed WGS alongside hybrid capture to determine integration of the BKPyV genome into the human genome. We could detect BKPyV in all cases, including different genotypes within tumor and adjacent normal tissues from the same patient, suggesting that multiple genotypic variants of BKPyV may be present within the tumor and/or matched normal samples.

We found that the BKPyV genome was integrated into the human genome in only six out of 19 (32%) UC specimens [Table 1] and ][Table S2]). Details of the integration sites, including the breakpoints in the BKPyV genome and human genomes, and other viral reads detected, are provided in [Fig. 1A], and [Tables S3 and S4]. These integration sites appear to be randomly distributed across the entire BKPyV genome, with average integration rates of 0.52, 0.50, 0.32, 0.37, 0.43, and 0.19 per 100 base-pair nucleotides for NCCR, agnoprotein, VP2, VP1, TAg, and tAg (small T antigen), respectively [Fig. 1B]. Among the six UC specimens, chromosome 8 and 12 exhibited a higher frequency of integration sites, with 5 and 4 sites, respectively [Fig. 1C]. Of these 21 sites, 76% were located in introns, 19% in intergenic regions, and only one breakpoint was identified in an exon [Table S3].

**Fig. 1:**
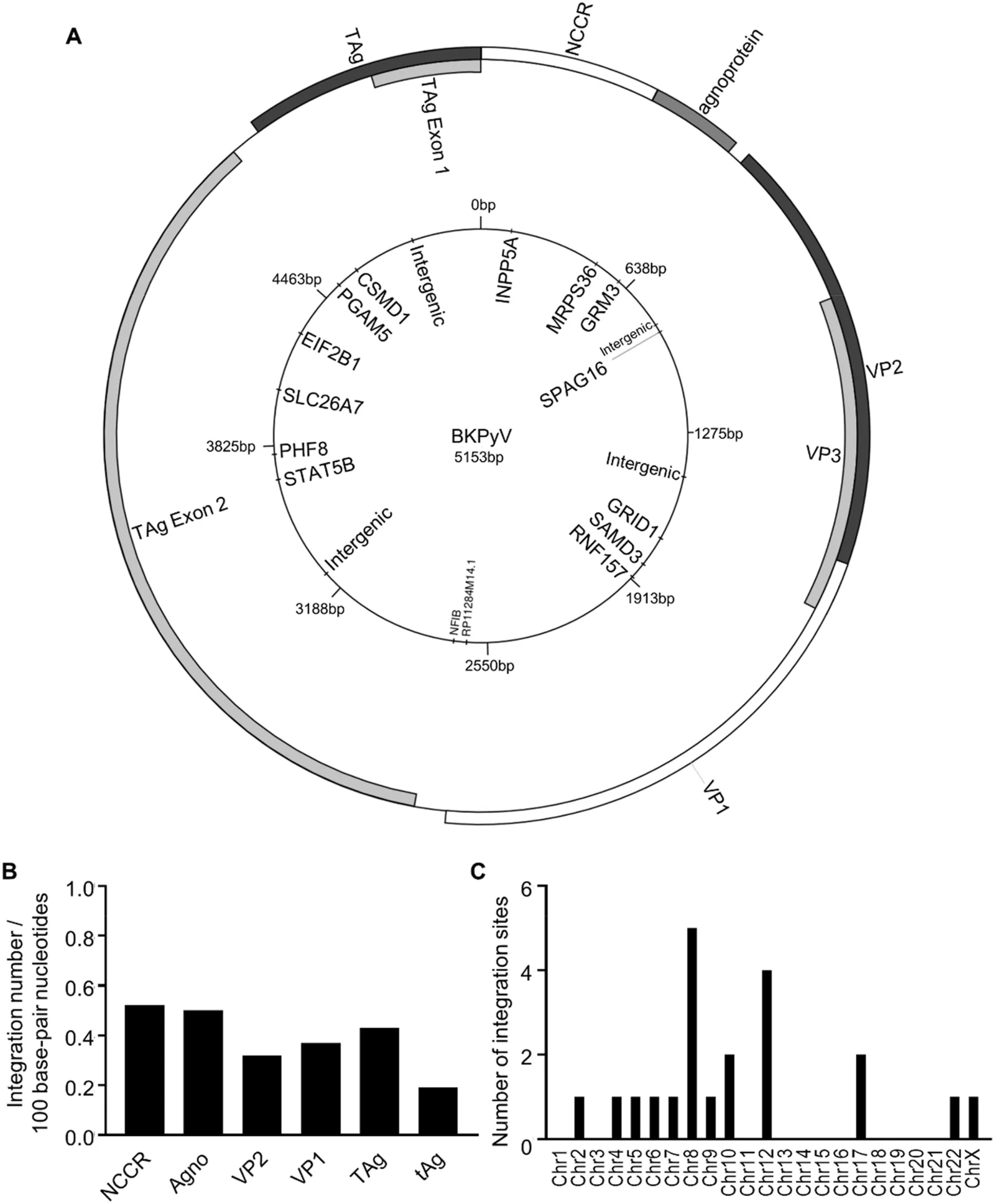
Distribution of BKPyV genome integration site breakpoints in the viral and human genomes. (A) The map displays the breakpoints of BKPyV genome integration sites at the viral genome, with letters indicating the corresponding breakpoints of the human genome. (B) The figure shows the number of integration sites per 100 base-pair nucleotides at different regions of the BKPyV genome. (C) The distribution of 21 BKPyV integration sites across the human chromosomes is displayed. The Y axis represents the number of integration sites, and the X axis indicates each of the chromosomes. NCCR: non-coding control region, Agno: agnoprotein, VP1: viral capsid 1, VP2: viral capsid 2, VP3: viral capsid 3, TAg: large T antigen, and tAg: small t antigen.

### 3.2 Verification of the BKPyV genome integration by Sanger sequencing

To validate BK polyomavirus (BKPyV) integration into the human genome as detected by WGS, Sanger sequencing was used to analyze fusion genes in two cases, L1 and L3. PCR was performed on genomic DNA extracted from urothelial carcinoma (UC) specimens of recipients L1 and L3. In case L1, a 5′ primer targeting the human *EIF2B1* gene and a 3′ primer targeting the BKPyV large T antigen (*TAg*) gene were designed. For case L3, a 5′ primer targeting the *TAg* gene and a 3′ primer targeting the human *SLC26A7* gene were used. PCR products of the expected sizes were purified following gel electrophoresis and subjected to Sanger sequencing. The sequencing results confirmed the WGS findings, revealing *TAg–EIF2B1* fusion in L1 [Fig. 2A] and *TAg–SLC26A7* fusion in L3 [Fig. 2B]. Based on sequence analysis, the fusion gene in L1 is predicted to encode a C-terminally truncated TAg protein fused to a 20–amino acid sequence derived from the *EIF2B1* intron, which contains a stop codon. In contrast, the fusion gene in L3 includes the BKPyV noncoding control region (NCCR) and early region with the *TAg* start codon, suggesting potential expression of an intact small T antigen (tAg) or a C-terminally truncated TAg.

**Fig. 2:**
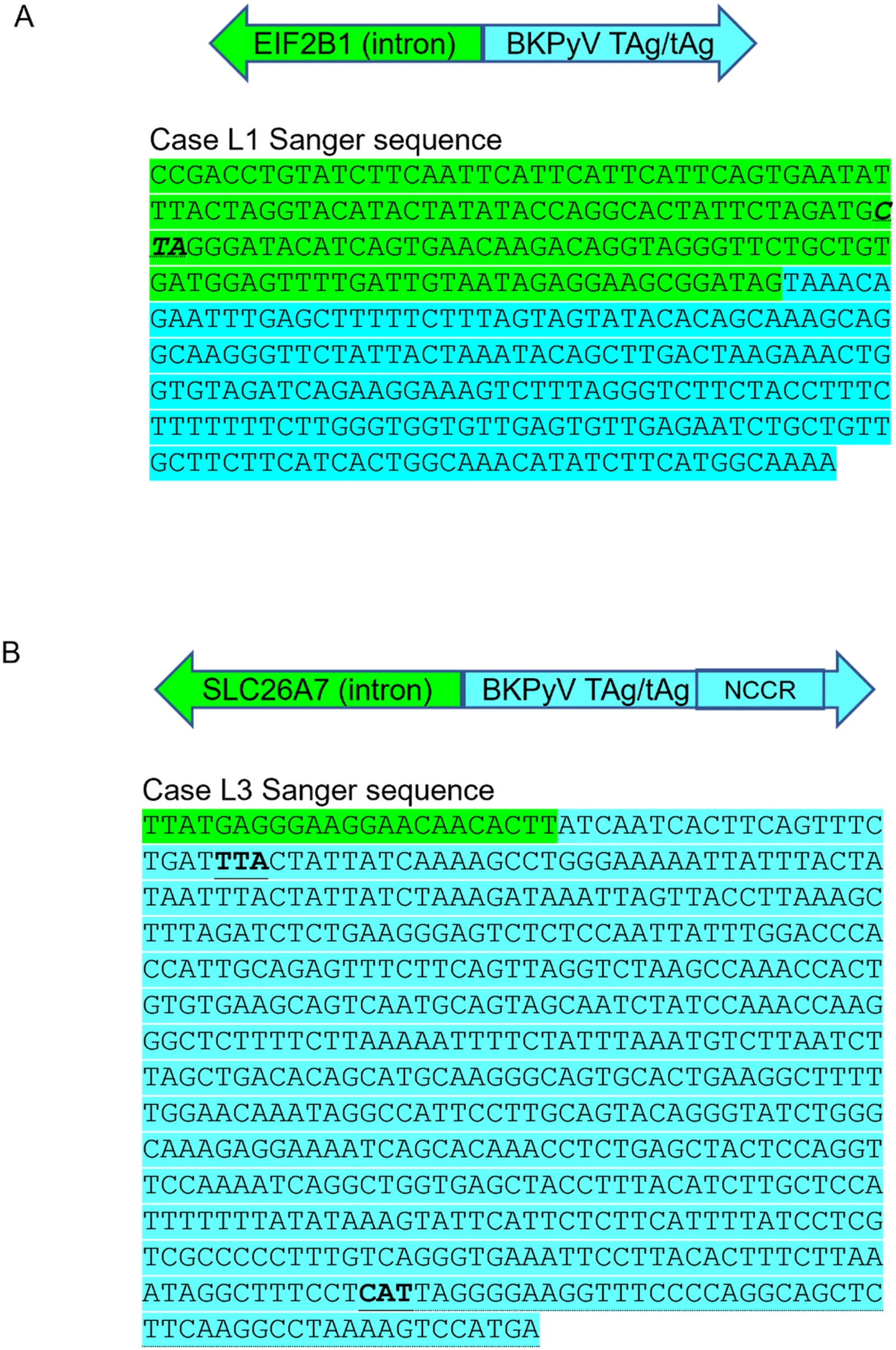
Confirmation of BKPyV genome integration by Sanger sequencing (A) PCR was performed on DNA extracted from the UC specimen of case L1 using a 5′ primer targeting the intron of the EIF2B1 gene and a 3′ primer targeting the BKPyV TAg gene, generating a predicted 361 bp product, verified by gel electrophoresis. The amplified fragments were subjected to Sanger sequencing. The EIF2B1 intron sequence is shown in green and the BKPyV TAg sequence in blue. A predicted stop codon (CTA) is indicated in bold. (B) For case L3, DNA was extracted from the UC specimen and PCR was performed using 5′ and 3′ primers annealing to the intron of SLC26A7 and the BKPyV TAg gene, respectively. The expected amplicon size was 635 bp, and gel electrophoresis confirmed a band at the predicted position. Sanger sequencing was conducted on the PCR product, with the SLC26A7 intron sequence highlighted in green and the BKPyV TAg sequence in blue. The start codon of TAg/tAg (CAT) and the stop codon of tAg (TTA) are shown in bold. The non-coding control region is underlined.

### 3.3 High prevalence of the AA mutational signature in UC specimens of kidney transplant recipients

In our previous study, we found a high frequency of the AA mutational signature, COSMIC signature 22 (SBS22), in non-transplant upper urinary tract UC and bladder cancers, which is characterized by a predominance of A > T transversions and more than 75% of A > T mutations occurring on the non-transcribed strand [27,28]. In the current study, the AA mutational signature was found in 16 of 19 UC specimens (84%) [Table 1]. In most UC specimens, the AA mutational signature exhibited the highest activity among the four analyzed COSMIC signatures [Fig. 3A]. Additionally, our UC specimens also displayed the common SBS5 that was present in almost all UC specimens with the AA mutational signature, albeit in low activity. Low activity of SBS45, indicative of sequencing artifacts or oxidative stress in cells, was detected in only two UC specimens. These findings confirm the high prevalence of the AA mutational signature in our UC specimens.

**Fig. 3:**
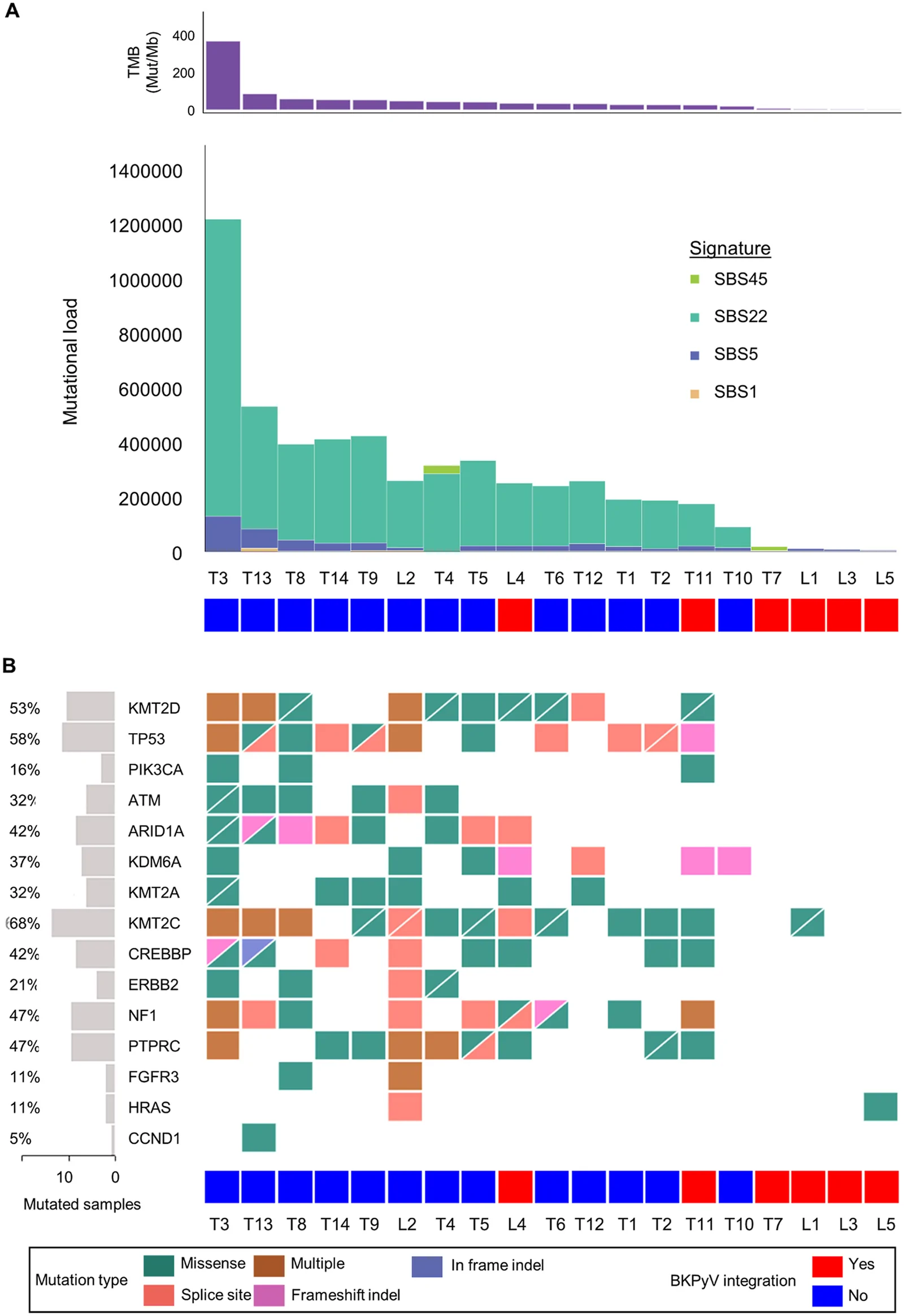
Tumor mutational burden, mutational signatures and BKPyV genome integration in UC. (A) The upper panel displays the tumor mutation burden (TMB) of 19 UC specimens, as analyzed by whole-genome sequencing. Stacked barplot showing the mutational load including non-synonymous mutation in each UC sample categorized against their mutational signature (middle panel). The x-axis represents the UC samples, while the y-axis indicates the mutational load in each sample, ranked by their SBS22 signature. The different colors represent the contribution of each mutational signature to the total mutational load, with the legend indicating the corresponding mutational signature for each color, including SBS1, SBS5, SBS22, and SBS45. The lower panel indicates the presence (red square) or absence (blue square) of BKPyV genome integration. (B) A heatmap is presented, which shows the frequency and mutational types of frequently mutated genes in each UC specimen.

Previous studies using WGS have identified several common gene mutations in UC, including those affecting genes involved in chromatin and cell-cycle regulation, as well as thereceptor tyrosine kinase (RTK) pathway [27,28,32,33]. In our study of 19 UC specimens, a high percentage of specimens had mutations in genes involved in chromatin modification, including *KMT2C* (68%), *KMT2D* (53%), *CREBBP* (42%), and *ARID1A* (42%) [Fig. 3B]. We also observed frequent alterations in genes involved in the cell-cycle pathway, including *TP53* (58%), *NF1* (47%), and *ATM* (32%), as well as mutations in genes involved in oncogenic signaling pathways, such as *ERBB2* (21%), *PIK3CA* (16%), *FGFR3* (11%), and *HRAS* (11%). These mutated genes identified in our 19 UC specimens were predominantly observed in UC specimens with the AA mutational signature, but were infrequently detected in UC specimens with BKPyV integration alone (T7, L1, L3, and L5). This suggests that distinct oncogenic mechanisms are involved in the development of UC in these two subtypes.

### 3.4 Contrasting mutation burdens in UC with BKPyV genome integration versus UC with the AA mutational signature

The UC samples were classified into two groups based on their AA mutational signature and BKPyV genome integration patterns. The first group demonstrated BKPyV genome integration with no or very low SBS22 activity (L1, L3, L5, and T7), while the second group showed significant SBS22 activity irrespective of BKPyV genome integration. The TMB in the first group (2.1 ± 0.8 mut/mb) was significantly lower than that in the second group (58.9 ± 21.6 mut/mb, *p* = 0.0005) [Fig. 4A]. Furthermore, the number of gene mutations in chromatin modification, cell cycle regulation, and the RTK-PI3K pathway was significantly lower in the first group compared to the second group (0.5 ± 0.3 versus 5.4 ± 0.6 per UC, *p* = 0.0008) [Fig. 4B]. In the first group, only a *KMT2D* missense mutation in L1 specimen and a *HRAS* missense mutation in L5 specimen were found [Fig. 4A]. These results indicate that UC with BKPyV genome integration and UC with the AA mutational signature have distinct mutation patterns and different oncogenic mechanisms.

**Fig. 4:**
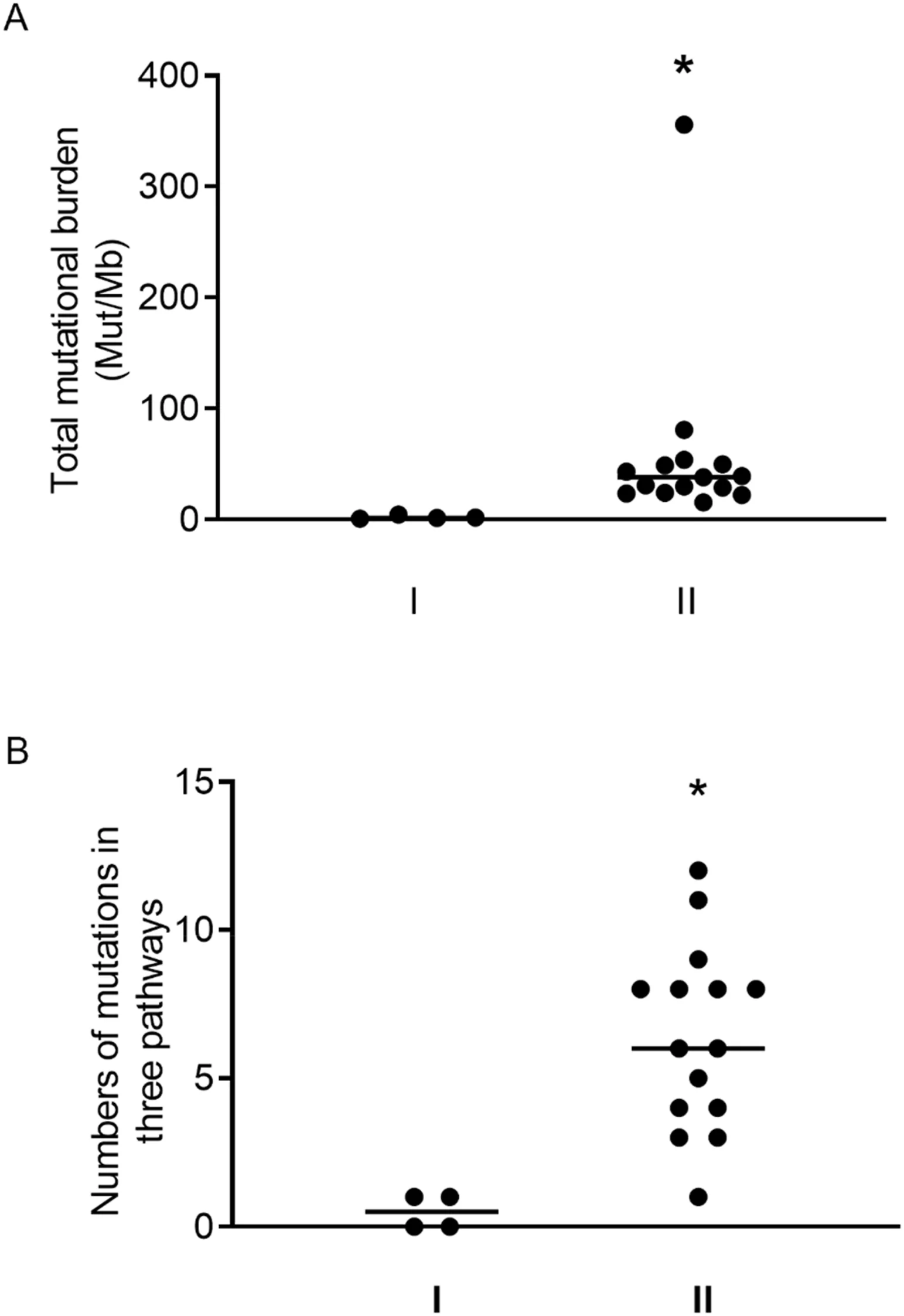
Comparison of mutation burdens in UC subtypes (A) The UC samples were classified into two groups based on their AA mutational signature and BKPyV genome integration. The first group included UC with BKPyV genome integration and no or very low SBS22 activity (L1, L3, L5, and T7). The second group had significant SBS22 activity, regardless of BKPyV genome integration (other specimens). (B) Comparisons of TMB and the number of mutations in three signaling pathways involved in chromatin modification, cell cycles, and the RTK-PI3K pathway between the two groups were analyzed using the Mann-Whitney test (* indicates p = 0.001).

### 3.5 Presence of APOBEC mutational signatures in UC specimens

Apolipoprotein B mRNA editing enzyme catalytic polypeptide (APOBEC) mutational signatures, specifically known as SBS2 and SBS13, are frequently observed in bladder cancers [31,34]. In this study, 89% (17 out of 19) of the specimens had either SBS2 or SBS3 (see Supplementary material, [Table S4]. Two specimens, L1 and L3, exhibited high activity of SBS2 and SBS13, while other specimens showed low or no activity [Fig. 5]. Notably, both L1 and L3, which contained BKPyV genome integration, were obtained from patients diagnosed with PyVAN [Table 2]. This suggests a potential link between APOBEC mutational signatures and BKPyV infection.

**Fig. 5:**
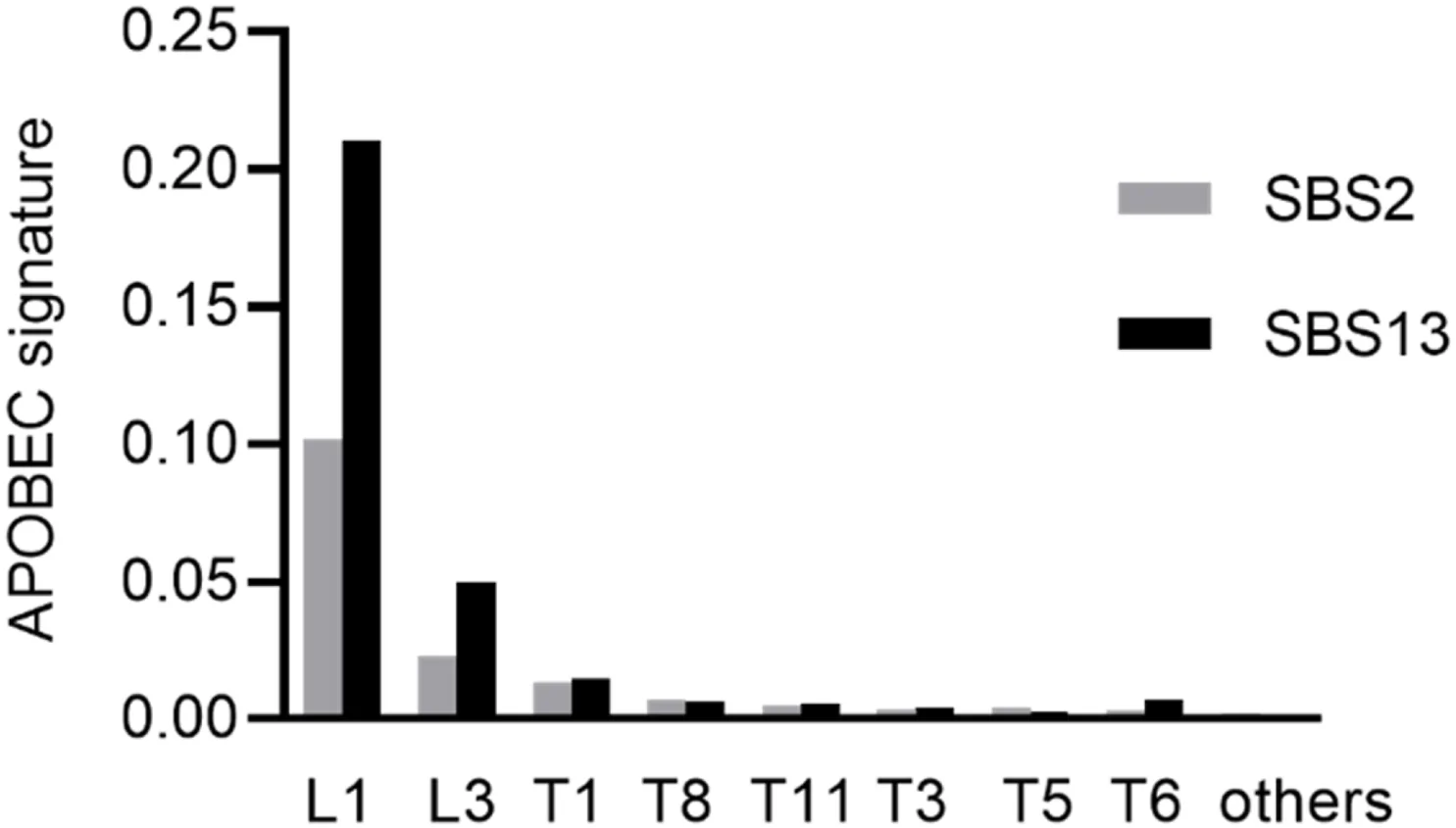
APOBEC mutational signatures in UC (A) Mutation signatures of Apolipoprotein B mRNA editing enzyme catalytic polypeptide (APOBEC), specifically SBS2 and SBS13, were analyzed in 19 UC specimens. The x-axis represents the UC specimens, while the y-axis indicates the mutational load, ranked by their APOBEC signatures. Only UC specimens with relatively high mutational loads are displayed; those with low or no mutational load are not shown.

## 4 Discussion

In this study, all 19 UC specimens were found to have either BKPyV genome integration and/or the AA mutational signature. Specifically, 6 specimens (32%) had BKPyV genome integration, while 16 specimens (84%) exhibited the AA mutational signature. Three specimens (16%) harbored both features [Fig. 6]. Several viruses, such as HBV, HPV, HTLV, and MCPyV, are considered oncoviruses capable of integrating their genomes into human DNA and causing cancer [32,33,35,36]. The integration of the BKPyV genome into the human genome was recently detected in UC and RCC specimens of kidney transplant recipients [[18], [19], [20], [21]]. However, the present study found a high proportion of UC specimens (32%) containing BKPyV genome integration. The proportion of positivity was even higher in fresh specimens with 4 out of 5 UC specimens affected (L1, L3, L4, and L5) containing BKPyV genome integration. In a recent study, 9 out of 43 (21%) UC specimens obtained from solid organ transplant recipients contained BKPyV genome integration [21]. Our study found a higher proportion of UC specimens with BKPyV genome integration, possibly due to several reasons including the use of fresh or frozen tissue specimens instead of FFPE blocks [35], variations in tumor content, sample purity, sequencing depth, or demographic differences.

**Fig. 6:**
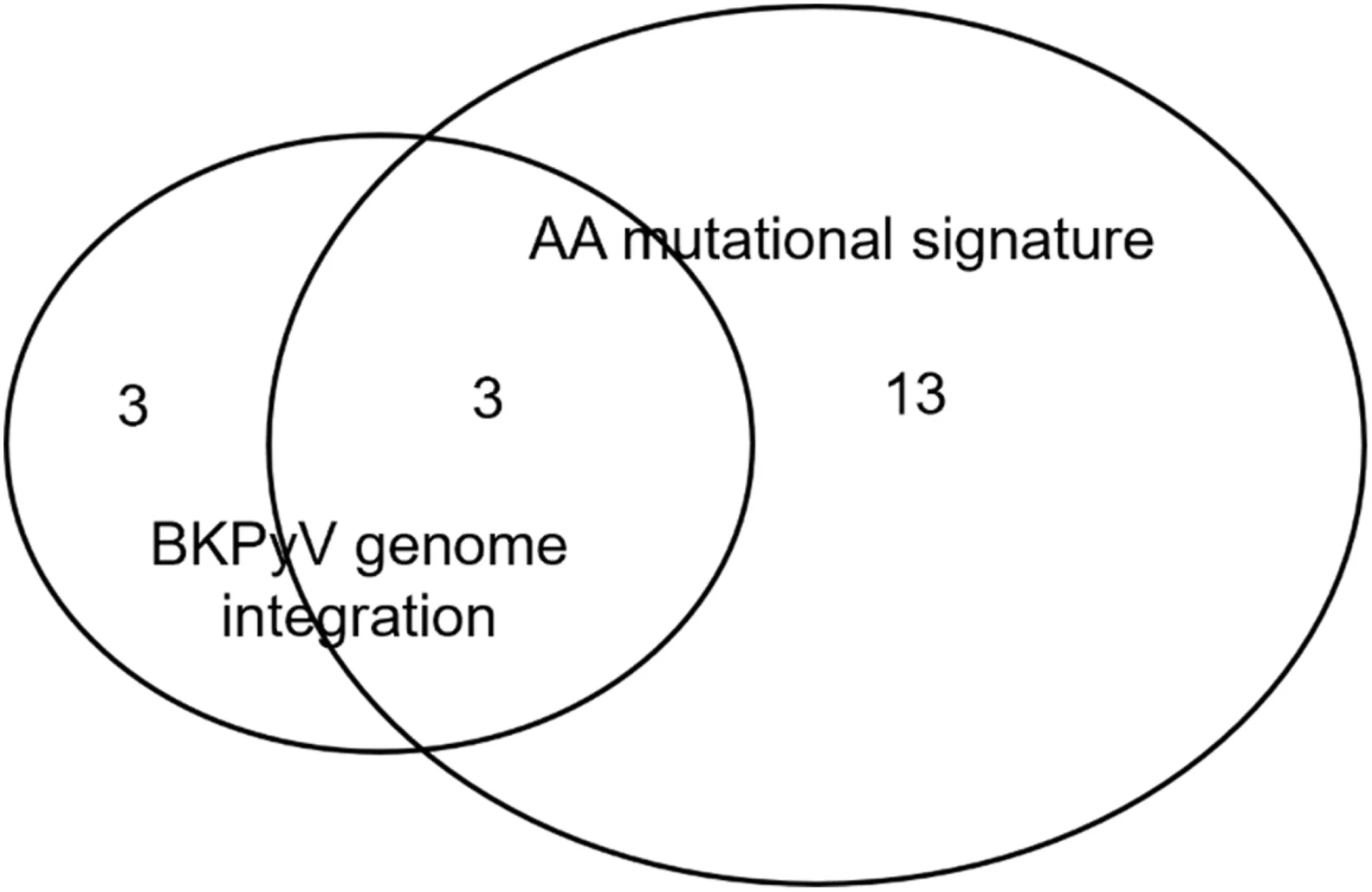
Summary of cases with BKPyV genome integration and/or AA mutational signature. Whole-genome sequencing was used to analyze the presence of BKPyV genome integration and the aristolochic acid (AA) mutational signature in 19 UC specimens. Results showed that 6 specimens contained BKPyV genome integration and 16 had the AA mutational signature, with 3 specimens showing both features. Only one specimen was found to be absent of these two features among the 19 UC specimens.

Kenan's study identified a single integration site with the entire BKPyV genome preserved and no disruption of tumor suppressor genes, suggesting that oncogenic transformation may be driven by overproduction of Tag [18,19]. In contrast, studies by Wang and Starrett reported multiple integration sites, implicating host gene disruption as a potential contributor to oncogenesis [20,21]. Consistent with these findings, our study also revealed multiple integration sites in UC specimens, although none were located within known tumor suppressor genes. In MCPyV-related MCC, non-random viral genome integration causes gene disruption and dysregulation, reprogramming host cellular signaling pathways, activating pro-cancer inflammation, and promoting oncogenesis [32,[37], [38], [39], [40], [41]]. In the Wang and Starrett studies, multiple integration sites potentially generate truncated TAg or VP1, if translated [20,21]. In MCPyV-associated MCC, viral genome integration often results in C-terminal truncations of TAg that promote tumor growth by inhibiting cell proliferation and regulating p53 [42,43]. Our predicted fusion products also suggest the generation of C-terminus-truncated TAg or intact tAg. Notably, in the L3 specimen, *BKPyV* integrated into the intron of *SLC26A7*, potentially producing an intact tAg protein. As tAg from MCPyV has been identified as an oncoprotein capable of inducing cellular transformation [44,45], we hypothesize that both intact tAg and truncated TAg derived from *BKPyV* integration may possess oncogenic potential.

Other possible explanations of BKPyV genome integration in cancer development include the disruption of host genes near proto-oncogenes or tumor suppressor genes, leading to their activation and unregulated cell growth. Furthermore, the integration process may induce DNA damage and chromosomal rearrangements, leading to genomic instability and generating additional mutations. The presence of integrated viral DNA can also trigger replication stress, further exacerbating genomic instability. Further verification is needed to determine the mechanism by which BKPyV genome integration contributes to UC development.

The APOBEC mutational signature is observed across various cancer types, suggesting APOBEC contributes to mutations in human cancers [31,34]. APOBEC3B overexpression and activation causes unexpected mutations by deaminating cytosines in the TpC context [31]. Our study revealed the presence of SBS2 or SBS13 in 17 of 19 UC specimens, consistent with the high prevalence of the APOBEC mutation signature in UC observed in other study [46]. Interestingly, two specimens (L1 and L3) displaying high APOBEC mutational signature activity were obtained from transplant recipients with PVAN. Induction of APOBEC expression following BKPyV infection through the RB binding motif (LXCXE) of the TAg gene has been documented [47,48]. The integration sequences in L1 and L3 preserved this motif. Our previous study demonstrated that patients with PVAN had early onset of UC development ^17^. We speculate that prolonged BKPyV infection facilitates APOBEC expression and subsequent UC development. Nevertheless, due to the lack of adequate controls, further investigation is needed to determine whether prolonged BKPyV infection facilitates APOBEC expression and subsequently contributes to UC development.

This study found an 80% prevalence of the AA mutational signature in UC, consistent with our previous finding of a 78% prevalence in hepatocellular carcinoma (HCC) in Taiwan [29]. Unlike Western countries and other Asian regions, where the prevalence is less than 50%, Taiwan shows a uniquely high rate of the AA mutation signature, likely due to high AA exposure [27,29,49].

In this study, we observe two types of mutation burdens in UC, with AA-associated UC having markedly more mutated oncogenes than UC with only BKPyV genome integration, indicating different mechanisms of carcinogenesis in these two types of UC. The TMB and the number of gene mutations was lower in BKPyV genome-integrated UC compared to UC with a AA mutation signature. In Starrett's study, they also reported a low mutation burden in BKPyV-associated tumors, in contrast to the high AA mutation burden observed in virus-free tumors of solid organ transplant recipients [21]. In MCPyV-related MCC, a low TMB was observed, while non-viral MCC had a large TMB [8]. In addition to the frame shift-mediated genomic aberrations caused by viral gene integration, MCPyV-encoded TAg and tAg play a crucial role in the development of MCC as they have been shown to possess oncogenic potential, even in truncated form [41]. We postulate that viral genome insertion-associated genomic aberrations and the production of oncogenic TAg and tAg may contribute to the development of BKPyV-genome integrated UC.

Somatic mutations associated with AA-induced UC have been reported to be highly prevalent in non-transplant population [49]. In this study, a high prevalence of mutations in chromatin modification genes, *KMT2C* (68%) and *KMT2D* (53%), and cell-cycle genes, *TP53* (58%), were observed in UC of kidney transplant recipients. Moreover, the prevalence of these mutations was even higher in AA-associated UC (*KMT2C*: 80%, *KMT2D*: 67%, and *TP53*: 73%). In AA-associated UC of the non-transplant population, Chen et al. reported a high prevalence of *TP53* mutation (56%) [49]. Lai et al. reported a higher prevalence of *TP53*, *KMT2D*, and *KMT2C* mutations in seven upper urinary tract UC samples from kidney transplant recipients [50]. These findings suggest that AA exposure causes DNA damage and the use of immunosuppressants impairs DNA repair, together leading to a high prevalence of gene mutations in UC of our kidney transplant recipients. Taken together, the screening of BKPyV integration and AA mutational signatures in the UC specimens could facilitate molecular risk stratification and provide individualized follow-up protocols and guide the tailoring of immunotherapy in kidney transplant recipients.

## 5 Conclusion

Our study provides evidence of a high prevalence of BKPyV genome integration, particularly in freshly prepared UC specimens, and a high prevalence of the AA mutation signature in UC of kidney transplant recipients. Distinct mutation features are present in BKPyV genome-integrated UC and AA-associated UC, indicating different mechanisms of carcinogenesis. These findings warrant further investigation in a large-scale study.

## CRediT authorship contribution statement

Jiun-Wen Wang: figure preparation and data analysis; Yi-Jung Li: conceptualization, data curation; Tzu-Hao Huang: resources and project administration; Hsin-Hsu Wu, Jason Yongsheng Chan, Jing Han Hong: review and editing; Hsu-Han Wang and Yang-Jen Chiang: resources; See-Tong Pang: conceptualization; Abner Herbert Lim: conduction of whole genome sequencing; Cedric Chuan Young Ng, Wei Liu, Jing Yi Lee: conduction of whole genome sequencing; Bin-Tean Teh: conceptualization, methodology; Ya-Chung Tian: conceptualization, roles/writing - original draft.

## Data availability statement

The data that support the findings of this study are available from the corresponding author upon reasonable request. The datasets supporting the conclusions of this article are available in the European Genome-Phenome Archive repository.

## Funding

This study was supported by grants (112-2314-B-182A-134-MY3) from the 10.13039/100020595National Science and Technology Council and grants (CMRPG3H0601, CMRPG3M1311, CORPG3P0101, and CMRPG3N1251) from the Chung Gang Medical Research Project to Dr Ya-Chung Tian.

## Declaration of competing interest

The authors declare no conflict of interest.
