# Supplementary material for: Distinct subtypes of kidney transplant-associated urothelial carcinomas harboring BK polyomavirus integration and aristolochic acid mutational signatures
Source: Biomed J. 2026 May 23;49(6):100990. doi: 10.1016/j.bj.2026.100990 (PMC13769077; doi:10.1016/j.bj.2026.100990)
Supplement: Supplementary file 1 [file mmc1.docx]

**A**

**B**

**C**

**D**

**E**

**Supplementary Fig. 1. Immunohistochemistry of urothelial carcinoma.** **A–D.** The left panels show hematoxylin and eosin (HE) staining, the middle panels display TAg immunostaining using anti-SV40 TAg (Merck Millipore, MABF121), and the right panels present VP1 immunostaining using anti-BK polyomavirus VP1 (Abnova, MAB3204-M19). The top images are captured at 100× magnification, while the bottom images are at 200× magnification. (A) Case L1, (B) Case L3, (C) Case L4, (D) Case L5. **E.** TAg immunostaining in cases T7 (left panel) and T11 (right panel) at 100× magnification (top) and 200× magnification (bottom). TAg immunostaining was strongly detected in cases L1 and L3, while in cases L4 and L5, it appeared scattered and patchy. In contrast, VP1 expression was minimal in these specimens. No TAg immunostaining was observed in cases T7 and T11.
