# Supplementary material for: Distinct subtypes of kidney transplant-associated urothelial carcinomas harboring BK polyomavirus integration and aristolochic acid mutational signatures
Source: Biomed J. 2026 May 23;49(6):100990. doi: 10.1016/j.bj.2026.100990 (PMC13769077; doi:10.1016/j.bj.2026.100990)
Supplement: Supplementary file 2 [file mmc2.docx]

Supplementary Table S1. Samtools alignment stats

| **ID** | **Status** | **Total sequences (Mil)** | **Mapped & paired (Mil)** | **Properly paired (Mil)** | **Duplicated (Mil)** | **Reads MQ0** | **Total bases (Gb)** | **Coverage (X)** | **Mapped bases (Mb)** | **Duplicated bases (Mb)** |
| --- | --- | --- | --- | --- | --- | --- | --- | --- | --- | --- |
| T1 | Neighboring | 594.44 | 593.56 | 590.90 | 137.51 | 15.27 | 89.17 | 29.72 | 88664.53 | 20626.39 |
| T1 | Tumor | 1156.86 | 1155.82 | 1151.46 | 414.12 | 29.93 | 173.53 | 57.84 | 172759.72 | 62117.99 |
| L4 | Neighboring | 569.05 | 565.74 | 538.81 | 85.87 | 23.00 | 85.36 | 28.45 | 77823.20 | 12881.12 |
| L4 | Tumor | 1409.12 | 1402.47 | 1329.23 | 240.15 | 51.59 | 211.37 | 70.46 | 196603.99 | 36022.49 |
| L5 | Neighboring | 585.99 | 585.44 | 582.63 | 117.11 | 11.86 | 87.90 | 29.30 | 87427.30 | 17567.25 |
| L5 | Tumor | 1200.67 | 1199.30 | 1193.70 | 288.56 | 23.44 | 180.10 | 60.03 | 179214.08 | 43283.89 |
| T3 | Neighboring | 585.34 | 584.49 | 582.17 | 104.23 | 13.75 | 87.80 | 29.27 | 87320.89 | 15633.76 |
| T3 | Tumor | 1216.56 | 1215.15 | 1206.31 | 294.22 | 27.19 | 182.48 | 60.83 | 181610.37 | 44132.37 |
| T5 | Neighboring | 689.97 | 688.85 | 686.27 | 136.59 | 13.59 | 103.50 | 34.50 | 102936.15 | 20488.13 |
| T5 | Tumor | 1486.06 | 1484.05 | 1476.49 | 445.13 | 30.10 | 222.91 | 74.30 | 221845.56 | 66770.03 |
| T6 | Neighboring | 718.64 | 714.48 | 687.18 | 122.28 | 24.13 | 107.80 | 35.93 | 101332.21 | 18342.02 |
| T6 | Tumor | 1345.49 | 1340.22 | 1271.41 | 207.13 | 36.72 | 201.82 | 67.27 | 192817.63 | 31069.72 |
| T7 | Neighboring | 651.51 | 650.68 | 648.13 | 180.16 | 14.86 | 97.73 | 32.58 | 97218.10 | 27024.05 |
| T7 | Tumor | 1478.65 | 1476.85 | 1471.48 | 568.13 | 31.78 | 221.80 | 73.93 | 220867.82 | 85219.57 |
| T7 | Tumor | 1430.56 | 1425.63 | 1328.16 | 232.64 | 44.26 | 214.58 | 71.53 | 206105.52 | 34896.12 |
| T14 | Neighboring | 620.19 | 617.90 | 596.14 | 77.52 | 16.96 | 93.03 | 31.01 | 89846.57 | 11627.33 |
| T14 | Tumor | 1128.55 | 1122.75 | 1084.29 | 165.91 | 36.61 | 169.28 | 56.43 | 160361.21 | 24886.26 |
| T9 | Neighboring | 500.17 | 498.24 | 478.89 | 59.65 | 15.01 | 75.03 | 25.01 | 70980.04 | 8947.48 |
| T9 | Tumor | 1087.05 | 1082.98 | 1001.69 | 127.13 | 29.73 | 163.06 | 54.35 | 153924.07 | 19070.08 |
| T10 | Neighboring | 616.50 | 615.68 | 612.94 | 126.22 | 11.78 | 92.48 | 30.83 | 91967.33 | 18932.68 |
| T10 | Tumor | 1186.48 | 1184.74 | 1156.59 | 251.96 | 24.03 | 177.97 | 59.32 | 176482.07 | 37793.60 |
| T11 | Neighboring | 358.61 | 356.50 | 338.63 | 47.12 | 12.01 | 53.79 | 17.93 | 50622.56 | 7067.52 |
| T11 | Tumor | 1397.50 | 1392.70 | 1295.89 | 219.59 | 42.55 | 209.62 | 69.87 | 199111.78 | 32938.57 |
| T12 | Neighboring | 739.37 | 736.53 | 718.63 | 112.09 | 19.13 | 110.91 | 36.97 | 108759.32 | 16814.20 |
| T12 | Tumor | 1429.69 | 1423.88 | 1395.30 | 215.09 | 38.25 | 214.45 | 71.48 | 209668.16 | 32263.49 |
| T13 | Neighboring | 605.01 | 604.39 | 602.01 | 106.18 | 14.41 | 90.75 | 30.25 | 90281.74 | 15927.54 |
| T13 | Tumor | 1202.85 | 1201.60 | 1197.67 | 309.46 | 27.31 | 180.43 | 60.14 | 179740.70 | 46419.36 |
| T14 | Neighboring | 596.96 | 596.28 | 593.95 | 100.77 | 11.86 | 89.54 | 29.85 | 89045.65 | 15115.87 |
| T14 | Tumor | 1188.78 | 1187.76 | 1184.10 | 272.19 | 25.32 | 178.32 | 59.44 | 177638.14 | 40828.36 |
| T15 | Neighboring | 602.38 | 601.68 | 597.85 | 98.83 | 11.90 | 90.36 | 30.12 | 89825.27 | 14825.18 |
| T15 | Tumor | 1213.73 | 1212.50 | 1207.62 | 293.10 | 25.10 | 182.06 | 60.69 | 181232.93 | 43964.87 |
| T2 | Neighboring | 655.93 | 653.38 | 634.05 | 90.30 | 15.49 | 98.39 | 32.80 | 96936.32 | 13545.38 |
| T2 | Tumor | 1454.60 | 1447.23 | 1293.70 | 199.51 | 35.64 | 218.19 | 72.73 | 214243.18 | 29926.44 |
| T4 | Neighboring | 590.19 | 587.80 | 545.02 | 90.96 | 24.35 | 88.53 | 29.51 | 76017.20 | 13644.63 |
| T4 | Tumor | 1628.39 | 1623.71 | 1520.76 | 237.90 | 54.63 | 244.26 | 81.42 | 233863.79 | 35685.05 |

**Supplementary Table S2.** BKV genotype and Virus Coverage

| **Case ID** | **Status** | **Genotype** | **Sequencing platform** | **Fraction of genome ≥ 5X** | **Mean Coverage of BKV Genome** |
| --- | --- | --- | --- | --- | --- |
| L1 | Neighboring | Undetermined | Whole Genome Sequencing | 0.00% | 0.0X |
| L2 | Neighboring | Undetermined | Whole Genome Sequencing | 0.00% | 0.0X |
| L3 | Neighboring | Undetermined | Whole Genome Sequencing | 0.00% | 0.0X |
| L1 | Tumor | Ic | Whole Genome Sequencing | 99.00% | 91.1X |
| L2 | Tumor | Undetermined | Whole Genome Sequencing | 0.00% | 0.1X |
| L3 | Tumor | IV | Whole Genome Sequencing | 98.00% | 110.8X |
| L4 | Neighboring | Ic | Hybrid Capture | 25.00% | 3.3X |
| L4 | Tumor | Undetermined | Hybrid Capture | 33.00% | 3.5X |
| L5 | Neighboring | Ic | Hybrid Capture | 97.00% | 23.4X |
| L5 | Tumor | Undetermined | Hybrid Capture | 79.00% | 9.8X |
| T5 | Neighboring | Undetermined | Hybrid Capture | 14.00% | 2.3X |
| T5 | Tumor | IV | Hybrid Capture | 0.00% | 0.1X |
| T6 | Neighboring | VI | Hybrid Capture | 23.00% | 2.8X |
| T6 | Tumor | VI | Hybrid Capture | 19.00% | 2.6X |
| T7 | Neighboring | Undetermined | Hybrid Capture | 100.00% | 72.6X |
| T7 | Tumor | IV | Hybrid Capture | 6.00% | 1.6X |
| T7 | Tumor | IV | Hybrid Capture | 100.00% | 286.7X |
| T8 | Neighboring | VI | Hybrid Capture | 100.00% | 289.6X |
| T8 | Tumor | IV | Hybrid Capture | 100.00% | 53.8X |
| T9 | Neighboring | IV | Hybrid Capture | 15.00% | 2.8X |
| T9 | Tumor | IV | Hybrid Capture | 63.00% | 7.8X |
| T10 | Neighboring | IV | Hybrid Capture | 99.00% | 20.0X |
| T10 | Tumor | IV | Hybrid Capture | 100.00% | 16.3X |
| T11 | Neighboring | V | Hybrid Capture | 84.00% | 26.5X |
| T11 | Tumor | Undetermined | Hybrid Capture | 17.00% | 2.0X |
| T12 | Neighboring | Undetermined | Hybrid Capture | 0.00% | 0.5X |
| T12 | Tumor | Undetermined | Hybrid Capture | 15.00% | 2.2X |
| T13 | Neighboring | V | Hybrid Capture | 78.00% | 8.1X |
| T13 | Tumor | IV | Hybrid Capture | 67.00% | 6.3X |
| T14 | Neighboring | V | Hybrid Capture | 100.00% | 303.8X |
| T14 | Tumor | Undetermined | Hybrid Capture | 100.00% | 277.9X |
| T15 | Neighboring | Undetermined | Hybrid Capture | 6.00% | 1.6X |
| T15 | Tumor | V | Hybrid Capture | 3.00% | 0.6X |
| T1 | Neighboring | IV | Hybrid Capture | 0.00% | 0.4X |
| T1 | Tumor | IV | Hybrid Capture | 0.00% | 0.1X |
| T2 | Neighboring | IV | Hybrid Capture | 1.00% | 0.5X |
| T2 | Tumor | IV | Hybrid Capture | 0.00% | 0.3X |
| T3 | Neighboring | IV | Hybrid Capture | 100.00% | 241.9X |
| T3 | Tumor | Undetermined | Hybrid Capture | 0.00% | 0.0X |
| T4 | Neighboring | IV | Hybrid Capture | 24.00% | 3.4X |
| T4 | Tumor | IV | Hybrid Capture | 0.00% | 0.8X |

| **Supplementary Table S3.** Summary of breakpoints and integration sites | | | | | | | | | | |  |
| --- | --- | --- | --- | --- | --- | --- | --- | --- | --- | --- | --- |
| **Sample (N)** | **Break point in BKPyV** | **Integration site in human** | **Tumor / Neighboring** | **Supporting reads** | **WGS / Hybrid Capture** | **Major Copy Number** | **Minor Copy Number** | **Total Copy Number** | **Status** | **Indels Detected spanning +/- 1kb** |  |
| L1 | TAg: 4289 | EIF2B1 intron (Chr12:124115229 - 124115289) | T | 2987 | Both | 2 | 0 | 2 | Neutral | Undetectable |  |
| L1 | TAg: 4535 | PGAM5 exon (Chr12:133298040 - 133298070) | T | 438 | Both | 2 | 1 | 3 | Gain | Undetectable |  |
| L3 | TAg: 4049 | SLC26A7 intron (Chr 8: 92303116 - 92303162) | T | 46 | Both | 3 | 2 | 5 | Amplification | Undetectable |  |
| L4 | NCCR: 122 | INPP5A intron (Chr10:134509463 - 134509406) | N | 4 | Hybrid Capture | 1 | 0 | 1 | Deletion | Undetectable |  |
| L4 | Agn: 485 | MRPS36 intron (Chr5: 68514904 - 68514843) | N | 3 | Hybrid Capture | 1 | 1 | 2 | Neutral | Undetectable |  |
| L4 | Intergenic: 2684 | NFIB intron (Chr9: 14215321 - 14215435) | N | 4 | Hybrid Capture | 1 | 0 | 1 | Deletion | Undetectable |  |
| L4 | VP1: 1837 | SAMD3 intron (Chr6: 130549272 - 130549179) | N | 4 | Hybrid Capture | 1 | 1 | 2 | Neutral | Undetectable |  |
| L4 | TAg: 4289 | EIF2B1 intron (Chr12:124115210-124115289) | N | 14 | Hybrid Capture | 1 | 1 | 2 | Neutral | Undetectable |  |
| L4 | Intergenic: 4626 | CSMD1 intron (Chr8: 4536112 - 4536011) | N | 5 | Hybrid Capture | 1 | 0 | 1 | Deletion | Undetectable |  |
| L5 | TAg: 4289 | EIF2B1 intron (Chr12:124115253-124115289) | N | 15 | Hybrid Capture | 1 | 1 | 2 | Neutral | Undetectable |  |
| T7 | VP1: 1903 | RNF157 intron (Chr17: 74218194 - 74218256) | T | 5 | Hybrid Capture | 3 | 1 | 4 | Amplification | Undetectable |  |
| T11 | Intergenic: 598 | GRM3 intron (Chr7: 86322907 - 86322840) | T | 7 | Hybrid Capture | 3 | 1 | 4 | Amplification | Undetectable |  |
| T11 | VP2: 825 | Intergenic (Chr 8: 77148747 - 77148864) | N | 2 | Hybrid Capture | 1 | 1 | 2 | Neutral | Undetectable |  |
| T11 | VP2: 857 | SPAG16 intron (Chr2: 214718887 - 214718784) | N | 2 | Hybrid Capture | 3 | 2 | 5 | Amplification | Undetectable |  |
| T11 | VP2: 1452 | Intergenic (Chr8: 20753050 - 20752944) | T | 4 | Hybrid Capture | 2 | 0 | 2 | Neutral | Undetectable |  |
| T11 | VP1: 1713 | GRID1 intron (Chr10: 87786943 - 87786852) | T | 5 | Hybrid Capture | 2 | 2 | 4 | Amplification | Undetectable |  |
| T11 | VP1: 2633 | RP11-284M14.1 intron (Chr4: 143882973 - 143882873) | N | 9 | Hybrid Capture | 2 | 1 | 3 | Gain | Undetectable |  |
| T11 | TAg: 3267 | Intergenic (Chr8: 58207410 - 58207317) | T | 2 | Hybrid Capture | 2 | 0 | 2 | Neutral | Undetectable |  |
| T11 | TAg: 3690 | STAT5B intron (Chr17: 40375866 - 40375753) | N | 3 | Hybrid Capture | 3 | 2 | 5 | Amplification | Undetectable |  |
| T11 | TAg: 3791 | PHF8 intron (ChrX: 53988989 - 53989049) | T | 1 | Hybrid Capture | 2 | 1 | 3 | Gain | Undetectable |  |
| T11 | tAg: 4878 | Intergenic (Chr22: 19631038 - 19630965) | T | 8 | Hybrid Capture | 2 | 0 | 2 | Neutral | Undetectable |  |
|  |  |  |  |  |  |  |  |  |  |  |  |
| Abbreviation of viral gene: Agn: agnoprotein, NCCR: non-coding control region, TAg: large T antigen, tAg: small t antigen, VP1: viral capsid 1, VP2: viral capsid 2. Abbreviation of human gene: CSMD1: CUB and Sushi multiple domains 1, GRID1: glutamate ionotropic receptor delta type subunit 1, GRM3: glutamate metabotropic receptor 3, EIF2B1: eukaryotic translation initiation factor 2B subunit alpha, INPP5A: inositol polyphosphate-5-phosphatase A, MRPS36: ribosomal protein S3, NFIB: nuclear factor I B, PGAM5: phosphoglycerate mutase 5, PHF8: PHD finger protein 8, RNF157: ring finger protein 157, SAMD3: Sterile Alpha Motif Domain Containing 3, SLC26A7: solute carrier family 26 member 7, SPAG16: sperm associated antigen 16, STAT5B: signal transducer and activator of transcription 5B | | | | | | | | | | |  |
|  |  |  |  |  |  |  |  |  |  |  |  |
|  |  |  |  |  |  |  |  |  |  |  |  |
|  |  |  |  |  |  |  |  |  |  |  |  |
|  |  |  |  |  |  |  |  |  |  |  |  |

| **Specimen** | **SBS2** | **SBS13** |
| --- | --- | --- |
| L1 | 0.10203 | 0.2107 |
| L2 | 0.0023 | 0.00055 |
| L3 | 0.02267 | 0.04996 |
| L4 | 0 | 0.00049 |
| L5 | 0 | 0 |
| T1 | 0.01348 | 0.01471 |
| T2 | 0.00201 | 0.00224 |
| T3 | 0.00366 | 0.0039 |
| T4 | 0.00148 | 0 |
| T5 | 0.00389 | 0.00291 |
| T6 | 0.00329 | 0.00676 |
| T7 | 0 | 0.00173 |
| T8 | 0.00684 | 0.00641 |
| T9 | 0 | 0 |
| T10 | 0 | 0.00313 |
| T11 | 0.00528 | 0.00572 |
| T12 | 0 | 0.00146 |
| T13 | 0.00169 | 0 |
| T14 | 0.00145 | 0.00151 |

**Supplementary Table S4.** Mutation load of APOBEC signatures (SBS2 and SBS13) in 19 UC specimens
